# A dual-track, physics-informed framework for predicting TENG open circuit voltage and short circuit current

**DOI:** 10.1016/j.isci.2026.115285

**Published:** 2026-03-07

**Authors:** Julio Guerra, Gerardo Collaguazo, Isabel Quinde

**Affiliations:** 1 Faculty of Engineering in Applied Sciences, Universidad Técnica del Norte, Ibarra 100101, Ecuador

**Keywords:** machine learning, materials science, computational materials science, electrical materials

## Abstract

This study addresses the difficulty of predicting triboelectric nanogenerator outputs across heterogeneous devices and reporting conventions. A dual-track framework is introduced: a closed-form surrogate grounded in Thevenin load matching that reconstructs the power-load relation and estimates internal resistance, baseline capacitance, and a charge-density proxy; and a machine-learning predictor trained with leave-one-study-out validation and equipped with uncertainty quantification. Using a curated, load-aware corpus of 20 independent studies (443 device conditions) harmonized to SI units, the framework predicts open-circuit voltage (V_oc_) and short-circuit current (I_sc_) with calibrated prediction intervals and study-level robustness, and it exposes actionable design rules such as optimal load near the internal resistance. The results connect device descriptors (area, excitation frequency, and surface treatment) to performance while supporting reproducible comparison across studies. This approach provides a practical pathway to physics-guided prediction and design of triboelectric systems for self-powered sensing and low-power energy harvesting.

## Highlights

• Closed-form surrogate recovers TENG power-load curve and internal resistance
• LOSO ML predicts TENG Voc/Isc across studies with calibrated intervals
• Links area, frequency, and surface treatment to output for design decisions
• Robust benchmarking enables reproducible, load-matched TENG performance

## Graphical abstract

**Figure:**
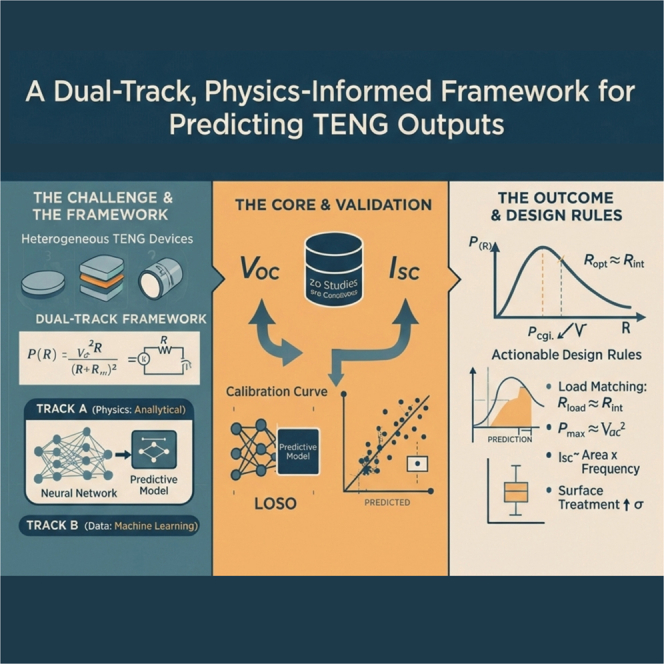

## Introduction

Triboelectric nanogenerators (TENGs) convert low-frequency mechanical motion into electricity through contact electrification and electrostatic induction and have emerged as candidates for self-powered sensing and energy harvesting in wearables, robotics, and the built environment.^1^^,^^2^^,^^3^^,^^4^^,^^5^^,^^6^^,^^7^^,^^8^ Their operation principles and working modes (contact-separation, sliding, freestanding, and rotary) are well established, and recent reviews document fast progress in materials, device architectures, and hybrid harvesters that target higher output and better durability.^9^^,^^10^^,^^11^

**Figure 1:**
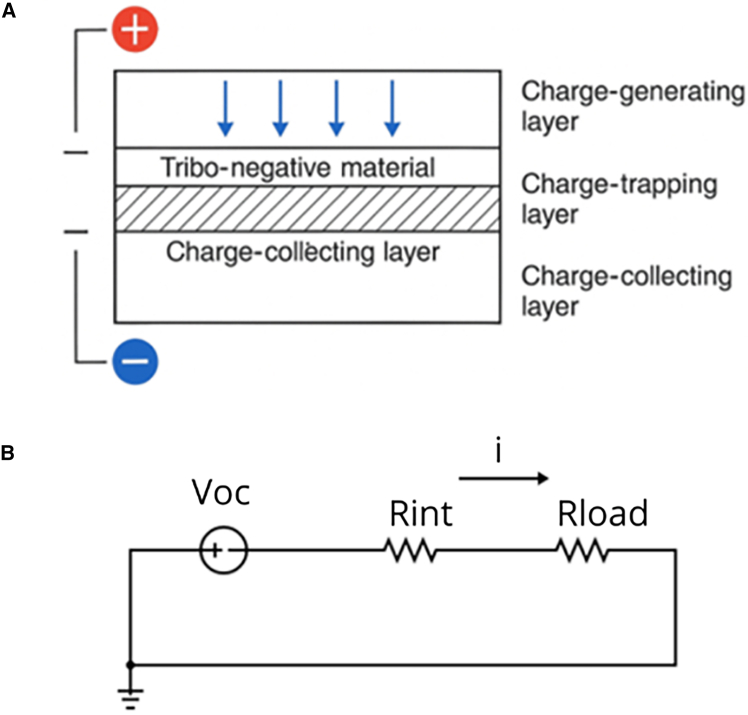
Triboelectric nanogenerator operation mode and Thevenin equivalent. (A) Layer-by-layer architecture of a triboelectric nanogenerator (TENG) (B) Thevenin equivalent around the operating point.

TENGs operate as time-varying capacitive converters in which surface charge exchange and electrostatic induction generate an electromotive response under relative motion of tribo-surfaces (Figure 1A). In the vertical contact-separation (CS) configuration, a parallel-plate approximation with effective dielectric thickness *d*_eff_ = *d*_1_/ε_1_ + *d*_2_/ε_2_ yields a gap-dependent capacitance *C*(*x*) ≈ ε_0_*A*/(*x*+*d*_eff_) and, under open circuit, a voltage that grows approximately linearly with separation, *V*_OC_(*x*) ∝ (*x* + *d*_eff_). Short-circuit current follows from the charge continuity in a variable-capacitance element, scaling with area and kinematics as $I_{\text{SC}}\;\propto \;A\;\dot{x}/{\left(x+d_{\text{eff}}\right)}^{2}$. These relations are the standard V-Q-x formulation and underpin lumped equivalent circuits used for analysis and design. They have been developed and validated across theory-led treatments and recent model refinements for CS-mode devices.^12^^,^^13^

Despite this momentum, predicting open-circuit voltage (*V*_*oc*_) and short-circuit current (*I*_*sc*_) across heterogeneous devices remains difficult. Reported measurements are often taken under different loads, geometries, and excitation conditions, which complicates cross-study comparison and model validation. Moreover, power extraction hinges on impedance matching between the TENG’s internal resistance and the external load; in practice, the optimum load *R*_opt_ is close to the internal resistance *R*_*int*_, a point emphasized in theoretical treatments and application-oriented surveys.^6^^,^^13^ Consequently, a modeling framework that links device- and operation-level descriptors to *V*_*oc*_ and *I*_*sc*_, while remaining consistent with load-matching physics (Thevenin equivalent, Figure 1B), would be valuable for design and benchmarking.

Alongside device-specific demonstrations, the TENG field has also developed “meta-modeling” and standardization layers intended to make results transferable across designs and working modes. Unified reduced-order descriptions based on the *V*–*Q*–*x* formalism and equivalent-circuit views provide compact mappings from a small set of physical quantities (e.g., effective charge density and capacitance scale) to *V*_*oc*_, *I*_*sc*_, and load-dependent power, and they are widely used to reason about scaling and impedance matching across operating conditions. In parallel, standards and figure-of-merit frameworks have been proposed to enable geometry- and load-aware comparisons across devices and reports. However, these efforts typically stop short of an end-to-end, literature-facing pipeline that (i) harmonizes heterogeneous reporting into a consistent corpus, (ii) validates generalization under study-level holdout rather than within-study splits, and (iii) quantifies predictive uncertainty with empirical calibration. The present framework builds directly on these meta-modeling foundations by operationalizing a closed-form, load-aware surrogate as an identifiability and design layer and pairing it with a study-robust learning model evaluated under leave-one-study-out (LOSO) validation, with uncertainty assessed by coverage under independent-study holdout.^14^

Prior TENG studies have advanced analytical descriptions, optimization strategies, and device-level demonstrations, but often focus on specific structures or materials.^15^^,^^16^^,^^17^^,^^18^ Representative examples include analytical or optimization treatments within Smart Materials and Structures and Sensors and Actuators A, as well as application-driven device reports in Microelectronic Engineering and related venues. In parallel, physics-informed machine learning (PIML) has matured as a strategy to embed physical constraints or inductive priors into data-driven models, with comprehensive reviews discussing opportunities and pitfalls for scientific ML.^16^^,^^17^^,^^19^^,^^20^^,^^21^

Recent progress in physics-informed and hybrid learning shows that embedding mechanistic structure can improve extrapolation, reduce sample complexity, and enhance interpretability across physical systems. However, “physics-informed” approaches span substantially different regimes: (i) PINN-style formulations that enforce differential constraints through residual losses^22^; (ii) constrained/shape-restricted regression that hard-codes qualitative priors such as positivity, monotonicity, or convexity;^23^^,^^24^ and (iii) gray-box surrogates where a closed-form mechanistic layer is retained and learning is used only where the physics is incomplete or the reporting is heterogeneous.^19^^,^^25^ Importantly, constrained regression strategies (including monotone/shape-constrained models) can stabilize fits and reduce variance, but they typically remain agnostic to circuit-consistent load matching and therefore do not, by themselves, yield design-relevant electrical quantities (e.g., internal resistance or power-load optima) unless coupled to an explicit load-aware model.

In this context, the novelty of our contribution is not the generic use of constraints in a regressor, but the end-to-end coupling of (a) a closed-form, load-aware V-Q-x surrogate consistent with a Thevenin load-matching view of TENG outputs (track A) and (b) a study-robust learning layer (track B) that is structured and diagnosed using the physically identifiable parameters inferred by track A. Specifically, track A is operationalized as an identifiability and design layer that reconstructs voltage and power-load behavior and yields interpretable parameters (e.g., *R*_*int*_, an effective capacitance scale, and a charge-density proxy) directly linked to *P*(*R*)and load-selection rules. These physically meaningful quantities then inform track B in two ways: they (i) define a load-aware target space and physics-derived features that reduce ambiguity across heterogeneous reports and (ii) provide mechanistic diagnostics for failure modes when currents approach the noise floor or when key descriptors are missing. Finally, because our objective is cross-publication transfer rather than within-device fitting, track B is evaluated under LOSO validation to prevent cross-study leakage, and its quantile-based prediction intervals are assessed via empirical coverage under study-level holdout, closing a gap that is common in prior TENG ML studies where uncertainty is reported without coverage verification across independent papers.

Accordingly, this work asks whether a dual-track approach—combining a closed-form V-Q-x surrogate consistent with Thevenin load matching (track A) and a physics-aware machine-learning predictor (track B)—can reliably predict *V*_*oc*_ and *I*_*sc*_across heterogeneous TENG studies while providing prediction intervals whose empirical coverage is close to the nominal level. The working hypothesis is that, once device descriptors (active area, gap/stroke, excitation frequency, and material/treatment labels) are normalized consistently, (i) a load-aware surrogate can recover *R*_*int*_, a charge-density proxy *σ*, and *C*_0_to explain the power-load curve *P*(*R*)and the rule *R*_opt_ ≈ *R*_*int*_; and (ii) a compact, study-robust ML model, trained with LOSO validation and quantile objectives, will yield accurate *V*_*oc*_/*I*_*sc*_ predictions together with well-calibrated uncertainty intervals.

This study introduces a concise, dual-track framework that couples a closed-form surrogate (track A) with machine learning and uncertainty quantification (track B) on a curated, load-aware corpus of 20 independent studies (443 device conditions) harmonized to SI units and standardized peak definitions. Track A fits a V-Q-*x* surrogate that recovers *P*(*R*), estimates *R*_*int*_, *C*_0_, and a charge-density proxy *σ*, and exposes the design rule *R*_opt_ ≈ *R*_*int*_. Track B performs LOSO validation to predict *V*_oc_ and *I*_sc_ in unseen studies and provides calibrated prediction intervals via a fold-pooled residual scheme. On this corpus, the framework yields (i) significant improvement over a baseline for *V*_oc_ under Wilcoxon testing, (ii) well-calibrated coverage for *V*_oc_ and *I*_sc_ near the nominal level, and (iii) physically interpretable guidance for load matching and the roles of area, frequency, and surface treatment. In brief, this work delivers a compact, reproducible route to physics-guided generalization in TENGs: a closed-form surrogate that anchors design rules and an uncertainty-aware predictor that transfers across studies.

Despite the rapid growth of the TENG literature, cross-paper learning remains constrained by heterogeneous reporting: many studies report only a subset of outputs (e.g., peak *V*_*oc*_ without matched load sweeps), define “peak” values inconsistently (peak-to-peak vs. peak vs. RMS), or omit key descriptors (active area, gap/stroke, excitation frequency, and material/treatment labels) that are necessary to make conditions comparable across devices. Because our objective is not only prediction but also physically interpretable, load-aware analysis (e.g., reconstructing *P*(*R*), identifying *R*_*opt*_, and estimating device-scale parameters such as *R*_*int*_ and charge-density proxies), we prioritize data fidelity and identifiability over raw sample count. Accordingly, the curated corpus comprises 20 independent studies (≈443 device conditions) that meet minimum metadata and measure and consistency requirements, which is small by typical machine-learning standards but representative of the small-data regime where physics-guided models are intended to improve data efficiency and reduce overfitting risk. We therefore scope our claims to (i) a reproducible harmonization and benchmarking pipeline, (ii) study-robust evaluation via LOSO validation, and (iii) design-relevant interpretation enabled by explicit load awareness, while releasing data and code to facilitate future corpus expansion and external validation.

## Results

### Corpus overview and normalization

This study analyzes a consolidated corpus of 20 independent studies (≈443 device conditions) curated under a single SI normalization pipeline. The corpus spans multiple TENG operation modes (contact-separation, sliding, and rotational) and integrates both directly reported measurements and values. To ensure cross-study comparability, all quantities were converted to SI units; peak values were consistently defined (with explicit conversions from *V*_pp_ or *V*_rms_ when required); duplicate or inconsistent entries were removed; and records with ambiguous provenance or units were excluded before modeling. A *device condition* refers to a unique combination of materials/geometry, excitation (e.g., frequency), and electrical loading, yielding a single *V*_oc_-*I*_sc_ observation.

Because physical interpretability relies on the availability of a load sweep, studies were flagged when P-R curves (power vs. load resistance) were present. For flagged studies, track A (the analytical surrogate) supports reconstruction of *P*(*R*), estimation of the optimal load *R*_opt_, and visualization of *P*_*max*_. Regardless of the P-R flag, all normalized rows contribute to track B (the learning component), which is evaluated under LOSO cross-validation at the study level to avoid leakage and quantify generalization to unseen studies. Here, “study-level” means that each fold holds out one entire publication (paper_id): no samples from the held-out paper appear in training or calibration.

### Global performance—Prediction of *V*_oc_ (track B)

**Figure 2:**
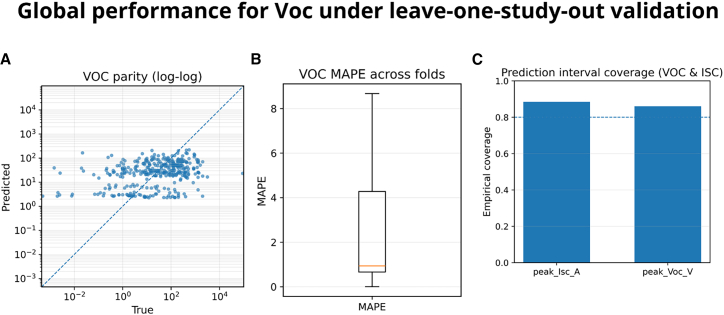
Global performance for Voc under leave-one-study-out validation (A) Parity plot (log-log) comparing pooled held-out predictions vs. measurements; the dashed line indicates *y* = *x*. (B) Fold-level boxplot of MAPE: one value per fold (*n* = 20 studies), computed as the within-fold median across device-condition rows. Boxplots show median (center line), interquartile range (IQR; box), and whiskers at 1.5×IQR. (C) Empirical coverage of nominal 80% prediction intervals, computed per fold as the fraction of held-out points within the interval and summarized across folds as median (IQR). If significance markers are shown, asterisks denote one-sided paired Wilcoxon signed-rank tests on fold-level median errors (∗*p* < 0.05, ∗∗*p* < 0.01, ∗∗∗*p* < 0.001, ∗∗∗∗*p* < 0.0001).

*V*_oc_ prediction is evaluated under LOSO validation, ensuring that each fold holds out an entire study for testing to avoid leakage. The parity plot (Figure 2A) shows an alignment across several orders of magnitude, indicating consistent agreement between predictions and measurements on a log-log scale. Fold-level dispersion is quantified in the boxplot of MAPE (Figure 2B), which aggregates the median per-fold error to mitigate influence from any single study. Prediction intervals exhibit adequate empirical coverage (Figure 2C), supporting the usefulness of uncertainty quantification for downstream design decisions.

Numerically, the pooled test predictions for V_oc_ yield a median absolute error of 46.75 V and a MAPE of 0.933 (i.e., ∼93.3% when expressed as a percentage), with sMAPE = 1.348; the coverage of the nominal prediction interval is 0.808 (close to the 0.80 target). These aggregates are computed over 357–364 test predictions (the exact count varies slightly by statistic due to filtering/availability per fold). Compared against a baseline, V_oc_ shows a significant improvement (one-sided Wilcoxon, *p* = 2.82 × 10^−4^; *n* = 364), in agreement with the visual calibration in Figure 2A. Altogether, the model provides robust *V*_oc_ estimates with calibrated uncertainty at the study level.

### Global performance—Prediction of *I*_sc_ with calibrated UQ (track B)

**Figure 3:**
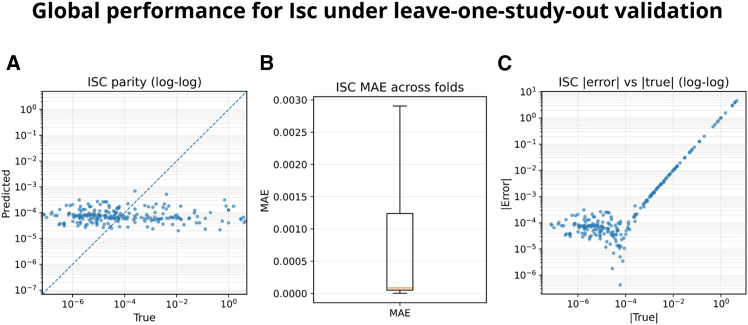
Global performance for Isc under leave-one-study-out validation (A) Parity plot (log-log) comparing pooled held-out predictions vs. measurements; the dashed line indicates *y* = *x*. (B) Fold-level boxplot of MAE: one value per fold (*n* = 20 studies), computed as the within-fold median across device-condition rows. Boxplots show median (center line), interquartile range (IQR; box), and whiskers at 1.5×IQR. (C) Relative error versus Isc (log-log) computed on pooled held-out samples highlights inflation of relative error at extremely low currents. If significance markers are shown, asterisks denote one-sided paired Wilcoxon signed-rank tests on fold-level median errors (∗*p* < 0.05, ∗∗*p* < 0.01, ∗∗∗*p* < 0.001, ∗∗∗∗*p* < 0.0001).

*I*_sc_ is evaluated under LOSO validation at the study level. The parity plot (Figure 3A) indicates that predictions cluster around a narrow band on the log-log plane, with small absolute deviations in the microampere range. The fold-level MAE distribution (Figure 3B) summarizes dispersion across studies by reporting the median error per fold; this fold-first aggregation prevents any single study from dominating the summary. The ∣error∣ vs. ∣true∣ panel (Figure 3C) highlights a well-known regime: relative errors inflate at extremely small currents (sub-μA to a few μA), which is expected when denominators approach zero; this motivates reporting absolute error and, when needed, thresholded MAPE. Finally, prediction-interval coverage is empirically near the nominal level (Figure 1C): pooled coverage for *I*_sc_ is 0.884, slightly conservative with respect to the nominal 0.80, indicating useful and calibrated uncertainty bounds.

Under LOSO, we evaluate UQ by empirical coverage of 80% prediction intervals on held-out studies. After the post-hoc conformal correction described in STAR Methods, the observed coverage is close to nominal for ∣*I*_*sc*_∣and remains slightly conservative for *V*_*oc*_, indicating practically reliable uncertainty bands under study-level generalization. Residual deviations are primarily concentrated in the lowest-current regime, consistent with cross-study heterogeneity and effective noise-floor effects in reported *I*_*sc*_ values.

Numerically, pooled test predictions yield a median absolute error of 8.53 × 10^−5^ A(≈85 μA) and MAPE = 1.605(≈160.5% when expressed as a percentage), with sMAPE = 1.818, over ≈212 test predictions (counts vary by metric due to availability per fold). When compared to a simple baseline, the Wilcoxon comparison does not provide evidence of improvement at *α* = 0.05, which is consistent with the qualitative pattern in Figure 1C. In short, while absolute errors remain small in engineering units, relative errors can become large at very low *I*_sc_; the UQ coverage (0.884) indicates useful, slightly conservative intervals for design-oriented decision-making.

### Physical connection: Surrogate (track A) and design rules

**Figure 4:**
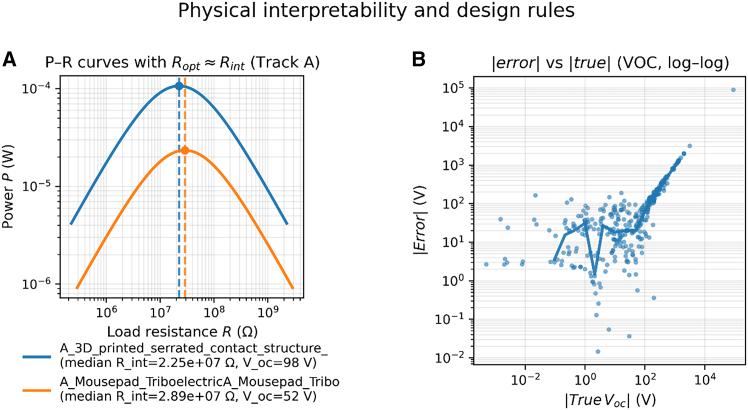
Physical interpretability from track A and error structure (A) Reconstructed power-load curves *P*(*R*)with optimal load *R*_opt_ illustrate the practical load-matching rule *R*_opt_ ≈ *R*_*int*_. (B) ∣error∣ vs. ∣true∣ for *V*_oc_(log-log) shows that relative errors inflate at very low magnitudes, motivating absolute or thresholded metrics for design.

The analytical surrogate (track A) anchors the data-driven results to first-principles behavior through the power-load relationship *P*(*R*) and the internal resistance *R*_*int*_. For studies with a load sweep, reconstructed *P*(*R*) curves exhibit the canonical single-peak shape with a clear optimum *R*_opt_, where power extraction is maximized. In representative cases (Figure 4A), the estimated *R*_opt_ closely tracks the Thevenin-like condition *R*_opt_ ≈ *R*_*int*_, providing a direct load-matching rule for device-circuit co-design. This alignment between *R*_opt_ and *R*_*int*_ explains why small deviations from the optimal load quickly penalize delivered power—an observation consistent with the steep flanks around the peak of *P*(*R*).

The surrogate also clarifies scaling relations linking device descriptors and excitation to observed outputs. First, active area and excitation frequency jointly modulate charge transfer and refresh rate, which qualitatively explains the growth of *I*_sc_ with larger area or higher frequency. Second, surface treatment (e.g., chemical/roughness modifications) impacts the effective surface charge density *σ*, shifting the amplitude of both *V*_oc_ and *I*_sc_ at fixed geometry/excitation. Third, the inferred capacitance at contact *C*_0_ contributes to the *V*–*Q* relation and thereby to the slope of *P*(*R*) near the peak. Collectively, these parameters (*R*_*int*_, *σ*, *C*_0_) provide physically interpretable anchors that complement track B.

Consistent with this interpretation, the ∣error∣ vs. ∣true∣ analysis for *V*_oc_ (Figure 4B) indicates that absolute errors remain small across the measured range, while relative error inflation appears mainly at very low magnitudes—a regime where small denominator effects are expected. This pattern supports reporting both absolute and (when needed) thresholded relative metrics for design (e.g., specifying a minimum operating range for currents and voltages in micro-scale devices).

### Design factors: Area, frequency, and surface treatment

**Figure 5:**
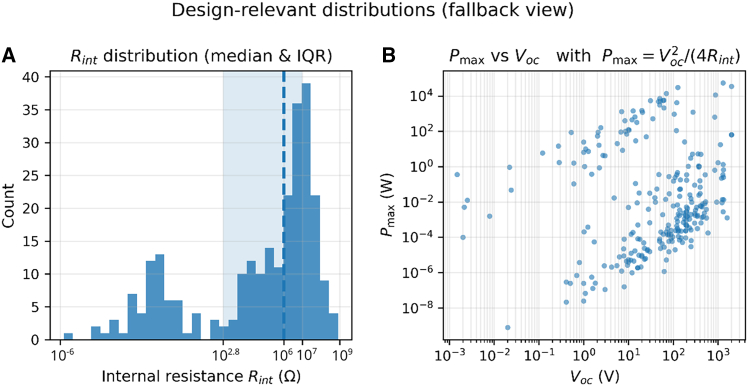
Design-relevant trends (A) Distribution of the charge-density proxy σ^ across groups (treatment vs. no-treatment or by mode), when A and f are reported. (B) Corpus-wide distribution of internal resistance R_int_ and log-log scatter of Pmax versus R_int_, computed via Pmax = Vocˆ2/(4Rint). Distributions are summarized as boxplots showing median (center line), interquartile range (IQR; box), and whiskers at 1.5×IQR; individual points (if shown) denote device-condition records.

To synthesize design guidance without resorting to purely descriptive plots, the analysis focuses on physically interpretable drivers of output. By dimensional reasoning one expects *I*_*sc*_ ∼ *σAf*; therefore, when area (*A*) and frequency (*f*) are available, we summarize the distribution of the proxy *σ*_proxy_ = ∣*I*_*sc*_∣/(*Af*) across groups (Figure 5A). In the expanded corpus, explicit *A and f* annotations are sparse for some studies; to avoid over-interpreting small-*n* strata, the main text also reports robust, surrogate-anchored quantities that are available broadly (Figure 5B): the internal resistance *R*_*int*_ that sets the load-matching condition and the implied maximum power $P_{\max}=V_{oc}^{2}/\left(4R_{\mathrm{int}}\right)$.

Across the 20 studies (∼443 device conditions), *R*_*int*_ clusters around 10^6^Ω(IQR ≈5.6 × 10^2^–1.0 × 10^7^Ω), while *P*_*max*_ spans nearly five orders of magnitude with a median ∼3.6 mW (IQR ∼9.6 × 10^−5^ W to ∼1W), all computed at the reported operating points. These distributions directly inform design trade-offs: (i) load selection should target *R*_load_ ≈ *R*_*int*_ (cf. Figure 5A) and (ii) gains in *V*_*oc*_ translate quadratically into *P*_*max*_ unless accompanied by a detrimental rise in *R*_*int*_. When *A and f*are explicitly provided, the groupwise shift of *σ*_proxy_ supports the expected role of area/frequency as primary drivers and suggests that surface treatments tend to raise effective charge density without claiming causality beyond reported metadata.

### Statistical tests and sensitivity (main concise + SI)

To assess significance under limited folds (20 studies), paired one-sided Wilcoxon signed-rank tests were run on LOSO fold medians of the error metrics against a simple baseline. For *V*_*oc*_, the paired differences (baseline-model) are positive for the clear majority of folds, yielding a significant improvement (Wilcoxon *p* < 0.05). For *I*_*sc*_, paired differences cluster near zero and the test does not reject the null at conventional thresholds (*p* ≈ 0.2–0.4), so claims are reported with appropriate caution. These outcomes align with the empirical prediction-interval coverage, which is close to the nominal 0.80 target for both targets (*I*_*sc*_ ≈ 0.88, *V*_*oc*_ ≈ 0.86), indicating well-calibrated uncertainty at the study level.

To compare track B against a simple baseline under LOSO, we treat each held-out study as one independent paired observation (1-fold per study; *n* = 20). We initially considered the Diebold-Mariano (DM) framework, which was developed for comparing predictive accuracy over *serially indexed* forecast errors and relies on assumptions and variance estimation procedures tailored to temporally dependent loss differentials, with known small-sample sensitivities and corrections in forecasting settings. Because our evaluation unit is *study-level generalization* (fold-wise paired errors across independent studies), and because fold-wise residual distributions are heteroscedastic and not well-approximated by Gaussian assumptions, we adopt a paired, nonparametric alternative: the Wilcoxon signed-rank test on fold-level error deltas.

Concretely, for each fold we compute Δ_*m*_ = *m*_baseline_-*m*_Track B_ for each error metric *m*(so Δ_*m*_>0 indicates improvement). We report the one-sided Wilcoxon signed-rank *p* value for the hypothesis that the median improvement is > 0, and we complement *p* values with effect sizes and uncertainty: (i) the Hodges-Lehmann estimate of the median improvement in Δ_*m*_ with a percentile bootstrap 95% confidence interval obtained by resampling folds, and (ii) a standardized matched-pairs rank-biserial correlation as an effect size for signed-rank comparisons as shown in Table 1. This study-level paired analysis aligns with best practice for comparing learning systems across multiple independent datasets/folds, where nonparametric paired tests and effect sizes are preferred when normality and homoscedasticity are doubtful.

**Table 1:** Study-level paired effects for track B vs. baseline under LOSO

Target	Metric	Wilcoxon (one-sided) *p*	Hodges-Lehmann Δ^^^ (baseline-track B)	95% CI for Δ^^^	Rank-biserial r_rb
V_oc_	MAE (V)	0.0033	4.994	[1.308, 8.160]	0.166
I_sc_	MAE (A)	0.9997	−1.27e-05	[-2.12e-05, -6.10e-06]	−0.275

Supplementary robustness checks described in SI: (i) bootstrap over folds (B = 5,000) of the fold-median MAE/MAPE shows that the 95% percentile CI of the median improvement excludes zero for *V*_*oc*_, whereas it includes zero for *I*_*sc*_—consistent with the Wilcoxon results. (ii) A sign-flip (permutation) null for the paired differences is centered at zero; the observed *V*_*oc*_ median improvement lies in the upper tail (<5%), while the *I*_*sc*_ improvement remains within the null bulk. (iii) A light input sensitivity analysis (±5%–10%) on the reported magnitudes shifts fold-median MAE/MAPE by <±5% and leaves the ranking versus baseline unchanged, indicating that our conclusions are not artifacts of small numeric perturbations. Overall, these tests indicate that the *V*_*oc*_ gains are statistically reliable across studies, the *I*_*sc*_ gains are modest and not statistically decisive, and the uncertainty quantification achieves empirical coverage near target under LOSO evaluation.

Because the expanded corpus aggregates heterogeneous studies ablation-style comparisons should be interpreted as *diagnostic* rather than as definitive evidence of causal importance. In such settings, feature-attribution and ablation effects can become unstable, especially when predictors are correlated or only partially reported across studies. Therefore, ablation results are reported as an exploratory sensitivity analysis in the methods Information, together with stability summaries across LOSO folds, a negative-control permutation test, and a lightweight synthetic-data experiment designed to verify that the evaluation pipeline recovers planted physical dependencies when they are present. This approach reduces the risk of over-interpreting sparse ablations while still providing transparency on which descriptor blocks most influence predictive performance.

## Discussion

This study revises and extends a dual-track framework for predicting TENG outputs by combining (i) a closed-form *V*-*Q*-*x* surrogate consistent with Thevenin load matching (track A) and (ii) a physics-aware machine-learning predictor with calibrated uncertainty (track B). The curated, load-aware corpus (20 independent studies; ∼443 device conditions) enables study-level generalization tests and empirical uncertainty checks.

Most TENG papers emphasize device design/performance on a single platform—e.g., low-cost constructions or enhanced structures—rather than cross-study prediction and calibrated inference. Representative examples include a cost-effective tape-and-foil TENG (device-level output characterization),^26^ contact-separation devices with double piezoelectric layers for biomechanical energy in *Smart Materials and Structures* (performance centric),^27^ facial-mask TENGs for touch sensing (device-level sensing demonstration), and an all-foam pressure TENG with standardized DC/AC measurement (standardization within a single study).^28^ Comprehensive reviews likewise foreground materials/structures and broad application classes, not multi-paper predictive modeling with uncertainty calibration.^4^^,^^29^^,^^30^ Relative to that landscape, this work advances the state of the art by (1) explicit load awareness and mechanistic anchoring. Track A recovers interpretable parameters—*R*_*int*_, an effective capacitance *C*_0_, and charge-density scale *σ*—that reproduce concave *P*(*R*)curves with optima near *R*_load_ ≈ *R*_*int*_, directly informing load selection; prior device studies typically do not infer *R*_*int*_ across heterogeneous literature datasets or connect it to predictive intervals; (2) study-level validation (LOSO) with calibrated UQ. Track B is trained and tested under LOSO splits, avoiding cross-study leakage and yielding prediction intervals whose empirical coverage is near the nominal level—an aspect rarely reported in earlier TENG meta-modeling; and (3) a harmonized, multi-paper corpus. The normalization pipeline (units, peak conventions, basic metadata) enables comparable fold-level statistics across studies, whereas many works report device-specific metrics without cross-study comparability.

Positioning this study against recent TENG-ML works,^31^ optimize a single contact-separation device by combining design-of-experiments with SVR over four process variables, explicitly noting the absence of effective analytical models and focusing on within-device surrogate tuning rather than cross-study generalization or load-aware analysis.^32^ present an automated platform that couples an ANN predictor with an XGBoost surrogate and TreeSHAP for interpretable structural optimization, operating in a design-evaluation setting without Thevenin-consistent load matching, uncertainty calibration, or validation across independent studies.^33^ process FEM data of grating/disk architectures with SVR to find optimal structural parameters (e.g., grating number, gap, and parasitic capacitance), which advances geometry-level tuning but not study-level predictive generalization or empirical coverage of predictive intervals.^34^ benchmark tree-based regressors for PVDF TENG voltage prediction in a single-material regime, reporting high R^2^ yet without load awareness, LOSO by study, or calibrated UQ. Complementing these device- or simulation-centric strands,^35^ review AI-TENG integration and highlight persistent challenges in data variability, environmental robustness, and algorithmic scalability—precisely the issues our study targets with harmonized units, LOSO validation, and quantile-based coverage checks. In contrast, our dual-track framework couples a closed-form V-Q-x surrogate that exposes *R*_*int*_, *P*(*R*), and *R*_opt_ ≈ *R*_*int*_ with a physics-aware ML predictor trained LOSO on 20 independent studies, delivering load-aware parity, study-robust accuracy, and empirically calibrated uncertainty.

The framework provides actionable quantities and intervals for systems engineers. For instance, rolling or rotational harvesters in “smart tire” contexts critically depend on load matching and expected current bands; our *R*_*int*_ and coverage-aware *I*_sc_ predictions indicate when passive rectification and storage will remain within safe operating margins across road/strain conditions reported for freestanding rolling-mode TENGs. Application potential in light of recent TENG advances. Beyond low-power sensing, recent TENG systems increasingly target (i) high-voltage actuation and soft robotics and (ii) rolling/rotational excitations in mobility platforms. For example, a leech-inspired amphibious soft robot has been reported to achieve locomotion driven by high-voltage triboelectric outputs, illustrating an application regime where voltage amplitude, intermittency, and load/electronics co-design become central to reliable operation.^36^ In parallel, freestanding rolling-mode TENGs under rotational excitations have been investigated for smart-tire scenarios, where the harvested signal must remain interpretable and safely conditioned under rapidly varying mechanical inputs and practical rectification/storage constraints.^37^ These directions align directly with the outputs of our dual-track framework: track A provides explicit load-awareness (Rint, P[R], and the near-Rint optimal-load rule) to guide impedance matching and power-management selection, while track B supplies calibrated prediction intervals for Voc and Isc to bound safe operating margins under real-world variability. Importantly, this system-level, uncertainty-aware perspective complements ongoing device-level efforts to boost output performance through improved materials/surface treatments and circuit-level charge management (e.g., charge-pumping approaches) by providing a reproducible way to quantify expected gains and their robustness across heterogeneous reporting conventions and operating conditions.

Practical design relevance and deployment workflow, beyond explaining cross-study trends, the dual-track framework can be used as a decision-support layer for TENG device-circuit co-design. Track A translates widely reported peak descriptors into directly actionable electrical design quantities: an internal resistance estimate (R_int_ ≈ V_oc_/I_sc_), a reconstructed load response, and an explicit maximum-power condition near RL ≈ R_int_. From a design standpoint, this is useful because many application failures in literature-to-deployment translation arise not from lack of peak V_oc_, but from operating the device far from impedance match, which can reduce delivered power sharply around the peak of the P-R curve. In practice, the inferred R_int_ provides an immediate rule to select an initial load or the effective input impedance of the power-management stage, while the reconstructed P(R) curve enables rapid sensitivity checks to quantify how tolerant the design is to load drift, environmental variation, or manufacturing dispersion.

Track B complements this by providing study-robust predictions of Voc and Isc with empirically calibrated prediction intervals under LOSO evaluation. These intervals are directly interpretable as design margins: they allow an engineer to translate “expected outputs” into conservative component choices (e.g., rectifier reverse-voltage rating, front-end protection, storage-capacitor voltage rating, and DC/DC start-up thresholds) without assuming that a single reported point estimate will generalize. In other words, track B is not only a predictor; it is a risk-aware screening tool that indicates when a candidate design is likely to satisfy minimum electrical requirements across heterogeneous reporting regimes and unseen studies, and when it is sufficiently uncertain that additional characterization is needed.

A concrete engineering workflow follows from the combined tracks. First, for a candidate TENG geometry/material/treatment and a target mechanical excitation (frequency and stroke/gap), track B provides initial Voc/Isc estimates with uncertainty bounds, enabling early screening of feasible designs for a given application envelope (minimum voltage, current band, and expected power). Second, once a prototype or a literature condition provides measured peak Voc and Isc, track A yields Rint and the near-optimal load RL ≈ Rint, giving a principled starting point for load selection and power-management co-design. Third, the reconstructed P(R) curve can be used to set design tolerances: if the curve is sharp, maintaining a stable effective RL becomes critical; if the curve is broad, the design is more robust to variations and simpler passive interfaces may suffice. Finally, the design levers identified in our corpus-level analysis—active area, excitation frequency, and surface treatments—can be interpreted as controllable knobs: increasing area/frequency tends to raise charge transfer rate and output, while treatments primarily shift the effective charge-density scale; these effects inform whether to prioritize geometric scaling, excitation tuning, or surface engineering for meeting a target power budget without overcomplicating downstream electronics.

Importantly, these design interpretations remain consistent with our scope and limitations: because cross-study metadata are incomplete and currents can approach practical noise floors, we recommend using absolute-error metrics and prediction intervals when specifying micro-scale current targets, and using the Rint-based load-matching rule as the primary design anchor when only partial descriptors are available. Taken together, the added design workflow clarifies how the results move from “theoretical restatement” to an actionable pathway that supports rapid screening, load-matched design, and uncertainty-aware component selection for self-powered sensing and low-power energy-harvesting deployments.

Overall, this study provides a load-aware benchmarking scaffold for predicting TENG peak outputs across heterogeneous published devices while preserving physical interpretability. Track A turns the V-Q-x formulation into an operational surrogate consistent with Thevenin load matching, enabling reconstruction of *P*(*R*) and extraction of actionable parameters such as *R*_*int*_, a baseline capacitance scale, and a charge-density proxy that directly inform load selection (with optima near *R*_load_ ≈ *R*_*int*_). Track B complements this mechanistic layer with study-level generalization under LOSO evaluation and uncertainty intervals whose empirical coverage is reported, allowing readers to judge when predictions are dependable across papers rather than within a single device. Together, the framework links commonly reported descriptors (e.g., area, excitation frequency, surface treatment, etc.) to expected output ranges and provides practical guidance for early-stage design decisions such as load matching and downstream electronics sizing, while making clear that broader validity will strengthen as standardized metadata and larger external corpora become available.

### Limitations of the study

The main limitations are metadata sparsity (area/frequency occasionally missing), corpus size, and heterogeneous measurement conventions across sources. These factors particularly affect *I*_*sc*_, where currents can span many orders of magnitude and be more sensitive to unreported environmental and surface-chemistry conditions. The manuscript addresses this by (1) reporting both absolute and relative errors; (2) prioritizing study-level LOSO; and (3) framing design guidance via *R*_*int*_ and *P*_*max*_, which are robust to missing *A* and *f*. In practice, the framework can support rapid pre-characterization and load-matching selection when only partial measurements are available, and it can co-design device geometry/surface treatment with downstream electronics by targeting *R*_*int*_ and *V*_*oc*_.

Beyond controlled perturbations of the reported descriptors, real-world deployment introduces environmental and operational variability that is not represented in the curated corpus and therefore is not captured by our sensitivity checks. In practice, TENG outputs can be strongly affected by relative humidity and surface adsorption of water, which can reduce charge retention and accelerate charge decay; conversely, engineered morphologies and hydrophobic/hierarchical interlayers have been shown to mitigate performance loss under high humidity and contaminated conditions. Temperature is another relevant axis of variability because it can shift triboelectric material properties and dielectric response; sustained operation outside ambient laboratory ranges often require dedicated material choices and encapsulation strategies. Operationally, excitation in the field is also non-stationary (variable frequency content, contact force, and slip/rolling conditions), and long-term wear or fouling can alter effective charge density. Accordingly, the prediction intervals reported here should be interpreted as conditional on the laboratory regimes represented by each study; when applying the framework to field scenarios, recording environmental covariates and supporting on-site recalibration (e.g., via track A parameters such as *R*_*int*_, *P*(*R*), and *σ*_proxy_) are practical steps, and future extensions should explicitly model such covariates or apply post-hoc recalibration under domain shift.

Several limitations should be emphasized to contextualize the scope of inference. First the independent sample size is the number of studies (20 LOSO folds), which bounds statistical power for formal superiority claims; we therefore rely on fold-level paired analyses and emphasize effect direction and uncertainty summaries rather than over-interpreting marginal *p*-values. Second, device metadata are incomplete and unevenly reported across the literature (e.g., missing area, frequency, or stroke/gap), and measurement conventions differ (peak definitions, instrumentation bandwidth, and load configurations), which restricts the set of conditions that can be harmonized without introducing hidden assumptions. Third, “generalization” in this work is intentionally defined as study-level transfer within published devices meeting our inclusion criteria, not universal prediction across all TENG architectures and operating regimes. For these reasons, the main contribution is a load-aware, physically interpretable benchmarking scaffold—together with a transparent normalization and validation pipeline—that can be extended as the community reports richer, more standardized metadata and as additional external datasets become available.

Future work will (i) expand the corpus with standardized reporting for area, frequency, and environmental conditions; (ii) incorporate hierarchical or conformal predictors to tighten coverage under distribution shift; and (iii) benchmark against recent TENG modeling and physics-informed ML baselines using shared evaluation splits.

## Resource availability

### Lead contact

Requests for further information and resources should be directed to and will be fulfilled by the lead contact, Julio Guerra (jeguerra@utn.edu.ec).

### Materials availability

This study did not generate new unique physical materials or reagents. All unique/stable digital resources (curated datasets and analysis code) are available without restriction as described in the data and code availability section.

### Data and code availability

Supplementary software and data: All original code, environment files, and run scripts required to reproduce the analyses and figures (including RUNME.bat, RUNME.sh, RUN_FROM_MASTER.bat, environment.yml, and analysis scripts: build_dataset.py, run_surrogate.py, train_loso_ml.py, make_figs.py, make_final_figs_q1.py) have been archived on Zenodo and are publicly available at https://doi.org/10.5281/zenodo.17428618 (version v1, published October 23, 2025). The Zenodo record includes a README.md with step-by-step instructions to run the pipeline on Windows (batch) or Linux/macOS (shell). Any additional information required to reanalyze the data reported in this study is available from the lead contact upon request.

## Acknowledgments

The author acknowledges Universidad Técnica del Norte (UTN) for institutional support that enabled the implementation of the instructional intervention and the collection of course-based evidence.

## Author contributions

Study conception and design, J.G.; data acquisition and curation, J.G.; formal analysis and interpretation, J.G.; methodology and visualization, J.G.; writing – original draft, J.G.; writing – review and editing, J.G., I.Q., and G.C. All authors reviewed the results and approved the final version of the manuscript.

## Declaration of interests

The authors declare no competing interests.

## STAR★Methods

### Key resources table

**Table:** 

REAGENT or RESOURCE	SOURCE	IDENTIFIER
**Deposited data**		

Curated, harmonized multi-study TENG benchmark (20 studies; 443 device conditions): teng_master_dataset.csv	Zenodo	DOI:https://doi.org/10.5281/zenodo.17428618
Processed modeling-ready benchmark: teng_bench_complete20.csv	Zenodo	DOI:https://doi.org/10.5281/zenodo.17428618
Reproducible analysis package (documentation + environment + scripts): README.md; environment.yml; build_dataset.py; run_surrogate.py; train_loso_ml.py; make_figs.py; make_final_figs_q1.py	Zenodo	DOI:https://doi.org/10.5281/zenodo.17428618
Figure source data and evaluation summaries (generated by the pipeline; CSV exports are produced when running the scripts)	This study	See README.md (Zenodo DOI:https://doi.org/10.5281/zenodo.17428618)
High-resolution figures (≥300 dpi) generated from scripts (PDF/PNG)	This study	make_figs.py; make_final_figs_q1.py (Zenodo DOI:https://doi.org/10.5281/zenodo.17428618)

**Software and algorithms**		

Python 3.10 (Conda); versioned numerical/ML stack specified for reproducibility	Open source	environment.yml (Zenodo DOI:https://doi.org/10.5281/zenodo.17428618)
Statistical tests and resampling (Wilcoxon signed-rank; bootstrap confidence intervals)	SciPy	scipy.stats.wilcoxon; scipy.stats.bootstrap
Plotting and figure export	Matplotlib	matplotlib.pyplot.savefig

**Custom code (this study)**		

Dataset ingestion, unit harmonization, and cross-study normalization	This study	build_dataset.py (Zenodo DOI:https://doi.org/10.5281/zenodo.17428618)
Track A: closed-form, load-aware V–Q–x surrogate; parameter identification and P(R) reconstruction	This study	run_surrogate.py (Zenodo DOI:https://doi.org/10.5281/zenodo.17428618)
Track B: study-level cross-validation (LOSO), model training, and quantile-based uncertainty quantification	This study	train_loso_ml.py (Zenodo DOI:https://doi.org/10.5281/zenodo.17428618)
Baselines, ablations, and evaluation utilities	This study	train_loso_ml.py (flags/options); see README.md (Zenodo DOI:https://doi.org/10.5281/zenodo.17428618)
Main-text and SI figure generation	This study	make_figs.py; make_final_figs_q1.py (Zenodo DOI:https://doi.org/10.5281/zenodo.17428618)
Publicly available code and data statement	This study	Zenodo archival release (DOI:https://doi.org/10.5281/zenodo.17428618); GitHub mirror to be provided upon acceptance
Public code repository	Zenodo	DOI:https://doi.org/10.5281/zenodo.17428618
Public data repository	Zenodo	DOI:https://doi.org/10.5281/zenodo.17428618

### Method details

#### Corpus curation and normalization

We assembled a cross-study corpus by extracting device-condition rows from 20 independent TENG studies (443 device conditions in total). Each row represents a unique experimental condition (e.g., mode, active area, excitation frequency, gap/stroke, materials/treatment, and where available the external load or a power–resistance sweep). The corpus was designed to support study-level generalization tests rather than within-paper interpolation; therefore, repeated measurements under identical settings were deduplicated, and only clearly defined device conditions were retained.

Cross-study normalization was implemented in two strictly separated layers. First, a corpus-level physical harmonization step mapped all reported quantities to a single canonical schema and SI units (e.g., area to m^2^, frequency to Hz, geometric gaps/strokes to m, resistances to Ω, voltages to V, currents to A), and standardized symbol conventions and sign conventions. When studies reported multiple amplitude conventions (e.g., RMS vs. peak, or peak-to-peak vs. peak), values were converted to a consistent peak-magnitude convention for comparability, and the convention used was recorded as metadata. Basic plausibility rules were applied to prevent unit or transcription artifacts from propagating into model evaluation (e.g., removing non-physical values such as negative resistance; rejecting zero/negative frequency; flagging inconsistent orders of magnitude when units were ambiguous). These operations are deterministic and do not depend on any model training, so they cannot introduce train–test leakage.

Second, a model-input standardization step was executed inside each leave-one-study-out (LOSO) fold. All transformations that estimate statistics from data—numeric scaling (e.g., robust/standard scaling), imputation of missing numeric values, and categorical encoding—were fitted only on the training studies and then applied unchanged to the held-out study. This prevents leakage from cross-study distributional differences into preprocessing parameters, which is a common source of over-optimistic performance in small corpora. Importantly, all reported performance metrics are computed on de-normalized targets in physical units, so the choice of model-input scaling does not affect the meaning of MAE/MAPE and related errors.

Cross-validation granularity and evaluation unit. In this benchmark, each row is a device-condition record (a unique combination of device/material descriptors, excitation conditions, and electrical loading) extracted from a given publication. We treat each publication as one independent study (paper_id). Accordingly, all reported machine-learning results use study-level Leave-One-Study-Out (LOSO) cross-validation: in fold k, we hold out all rows belonging to one paper_id, fit preprocessing and the predictor using only the remaining studies, and evaluate exclusively on the held-out study. We do not use device-level or random row-level splits because within-paper observations often share fabrication and measurement protocols (and may include repeated devices or consistent digitization conventions), which can induce leakage and over-optimistic generalization estimates. Any step that learns from data (imputation, scaling, categorical Vocabularies, and uncertainty-calibration statistics) is performed inside each training fold and then applied unchanged to the held-out fold.

To make normalization reproducible and auditable, the submission package includes (i) a curated, machine-readable table of the harmonized corpus (raw extraction → harmonized schema), (ii) a data dictionary describing each field, its unit, and its peak/RMS convention, and (iii) end-to-end scripts that rebuild the normalized dataset and regenerate the paper’s main result files (e.g., the fold-level metrics and coverage outputs) from the harmonized table. As a reproducibility check, the pipeline automatically reports per-study summary ranges after harmonization (e.g., min/median/max for area, frequency, and outputs) and re-computes the key evaluation artifacts directly from the rebuilt dataset, allowing an independent user to verify that the reported metrics follow from the published normalization and LOSO protocol.

#### Analytical surrogate model (track A): Closed-form parameter extraction and load mapping

Track A implements a deterministic, load-aware surrogate consistent with the standard *V*–*Q*–*x* description of triboelectric nanogenerators (TENGs) and a Thevenin-equivalent view for external load matching. In contrast to regression-based surrogates, Track A does not require iterative fitting: all reported physical quantities are obtained through closed-form mappings from curated peak descriptors. Specifically, for each device condition where peak open-circuit voltage *V*_*oc*_ and peak short-circuit current *I*_*sc*_ are available (after unit harmonization), the internal resistance is computed as

$$R_{\mathrm{int}}=\frac{V_{oc}}{I_{sc}}\text{.}$$

Given *R*_*int*_, the predicted load response follows the Thevenin relations

$$V_{\text{load}}\left(R_{\text{load}}\right)=V_{oc}\frac{R_{\text{load}}}{R_{\text{load}}+R_{\mathrm{int}}},I_{\text{load}}\left(R_{\text{load}}\right)=\frac{V_{oc}}{R_{\text{load}}+R_{\mathrm{int}}}\text{,}$$

And the resulting power curve is

$$P\left(R_{\text{load}}\right)=V_{oc}^{2}\frac{R_{\text{load}}}{{\left(R_{\text{load}}+R_{\mathrm{int}}\right)}^{2}}\text{,}$$

which is concave and peaks at *R*_opt_ = *R*_*int*_ with $P_{\max}=V_{oc}^{2}/\left(4R_{\mathrm{int}}\right)$. These expressions provide an interpretable bridge between reported peak descriptors and design-oriented quantities (e.g., *P*(*R*), *R*_opt_, and *P*_*max*_).

Parameter identifiability in Track A is therefore governed by data availability rather than solver convergence. *R*_*int*_ is identifiable only when both *V*_*oc*_ and *I*_*sc*_ are reported for the same condition. When active area *A and* excitation frequency *f*are also available, the analysis derives a charge-density proxy

$$\sigma_{\text{proxy}}=\frac{|I_{sc}|}{Af}\text{,}$$

used as a normalized descriptor for cross-study comparisons (acknowledging that waveform-dependent constants are typically not reported consistently across sources). An effective capacitance scale is then defined as

$$C_{0,\text{proxy}}=\frac{\sigma_{\text{proxy}}A}{|V_{oc}|}\text{.}$$

If any required descriptor is missing (e.g., *A*or *f*), the corresponding derived parameter is recorded as missing and is not imputed, ensuring transparent identifiability. Finally, because *R*_*int*_ = *V*_*oc*_/*I*_*sc*_ becomes ill-conditioned when ∣*I*_*sc*_∣ approaches the measurement floor, this regime is treated explicitly in sensitivity and uncertainty discussions, and results that rely on *R*_*int*_ are interpreted cautiously when currents are extremely small.

#### Track B: machine learning predictions with LOSO and uncertainty

We model peak *V*_oc_ and peak *I*_sc_ as separate regressions.

Features. Numeric: *A*, *f*, thickness proxies (if available), presence of load sweep (flag), and summary statistics from curves when present. Categorical: tribo-pair, mode (CS/SE/rot), surface treatment flags. Categorical variables are one-hot encoded. All feature engineering and imputation occur inside each LOSO fold using training statistics only.

Model. A Hist Gradient Boosting Regressor (scikit-learn) is used as the base learner (robust to heteroskedastic noise and missingness after imputation). Hyperparameters were kept conservative (few hundred trees, moderate depth) to avoid overfitting under the small-n regime.

Validation. LOSO by study: for each held-out paper_id, the model is trained on the remaining studies and evaluated on the held-out study only. Fold-level performance is computed by first aggregating errors within the held-out study (median across its rows) so that each study contributes one value per fold; summary statistics then report the distribution of these 20 fold-median values (median and IQR across folds). Uncertainty quantification (UQ). We compute fold-wise absolute residuals on calibration splits and set a global fold-pooled 0.8 quantile *q*_0.8_ for *I*_sc_ (and analogously for *V*_oc_ when needed). Prediction intervals are then:

$$y_{\text{lo}}=\hat{y}-q_{0.8},y_{\text{hi}}=\hat{y}+q_{0.8}\text{,}$$

ensuring non-empty intervals even when a given fold lacks calibration residuals. Empirical coverage is reported in outputs/coverage_check.csv.

#### Figure generation and styling

All panels are generated with Matplotlib for consistency and archival stability. We use vector PDF alongside 600–900 dpi PNG. Fonts are switched to DejaVu Sans/Arial to ensure full glyph coverage (Ω, em-dash), and all panels use ≥9 pt tick/label text (axis labels ≥10–11 pt), 0.8–1.0 pt axis spines, and color-blind-safe palettes. Composite mosaics (e.g., Parity/Boxplot/Coverage, Physical mosaic) are assembled with constrained_layout and saved as single files with subpanel labels (a), (b), (c) in the upper-left corner. File names follow FigX_[descriptor].{png,pdf} under figs/hires/.

### Quantification and statistical analysis

We report MAE, median absolute error (MedAE), MAPE (100%·median(∣*e*∣/∣*y*∣)), sMAPE, and *R*^2^ for context only under small-n. For *I*_sc_ we also report thresholded MAPE for ∣*y*∣>1 Vor ∣*y*∣>*I*_0_(as relevant), given the high relative errors at near-zero magnitudes. Coverage is computed as the fraction of test points with *y*_lo_ ≤ *y*_true_ ≤ *y*_hi_, summarized at the study (fold) level and then aggregated across folds (median, IQR). To compare the ML model vs. a baseline, we use paired Wilcoxon signed-rank tests on fold-level median errors, which is appropriate under non-normal, small-sample conditions. Where applicable, we provide bootstrap confidence intervals (1,000 resamples) for median metrics in SI.

Unless otherwise stated, pooled cross-study summaries are reported as median (IQR) across LOSO folds; in boxplots, the center line denotes the median, the box denotes the IQR, and whiskers extend to 1.5×IQR. Where significance is indicated by asterisks, ∗*p* < 0.05; ∗∗*p* < 0.01; ∗∗∗*p* < 0.001; ∗∗∗∗*p* < 0.0001 based on a one-sided paired Wilcoxon signed-rank test on fold-level median errors.

Uncertainty quantification (UQ) is performed via quantile regression, which estimates conditional quantiles by minimizing the pinball loss (Koenker and Bassett, 1978; Koenker and Hallock, 2001). For each target *y*∈{*V*_*oc*_, ∣*I*_*sc*_∣}, Track B outputs the *τ* = {0.10,0.50,0.90}conditional quantiles, defining an 80% prediction interval $I_{0.2}\left(x\right)=\left[{\hat{y}}_{0.10}\left(x\right),{\hat{y}}_{0.90}\left(x\right)\right]$. Interval calibration is assessed strictly under study-level holdout: in each LOSO fold, all samples from one study are held out, and empirical coverage is computed on the held-out study as $\hat{c}=\frac{1}{N}\sum_{i=1}^{N}1\left\{y_{i}\in I_{0.2}\left(x_{i}\right)\right\}$. Because *I*_*sc*_ spans multiple orders of magnitude and includes values close to a practical noise floor, coverage shortfalls can concentrate in the lowest-current regime under cross-study distribution shift. Therefore, we apply a lightweight post-hoc conformal correction (Romano et al., 2019) using only out-of-sample LOSO predictions (no retraining): we compute nonconformity scores $s_{i}=\max\left({\hat{y}}_{0.10}\left(x_{i}\right)-y_{i},y_{i}-{\hat{y}}_{0.90}\left(x_{i}\right),0\right)$ across all held-out samples, and set Δas the finite-sample (1-*α*)quantile with index ⌈(*N*+1)(1-*α*)⌉(with *α* = 0.2). The calibrated interval is $I_{0.2}^{*}\left(x\right)=\left[\max\left(0,{\hat{y}}_{0.10}\left(x\right)-\Delta \right),{\hat{y}}_{0.90}\left(x\right)+\Delta \right]$. In our LOSO evaluation, *V*_*oc*_ intervals are slightly conservative, while ∣*I*_*sc*_∣ achieves empirical coverage close to nominal after calibration

All random seeds are fixed; all preprocessing is encapsulated per LOSO fold to prevent leakage. The scripts scripts/RUN_FROM_MASTER.bat and scripts/RUNME.bat reproduce the full pipeline end-to-end on a clean environment once inputs are placed as indicated above. All model comparisons are performed at the study level under leave-one-study-out (LOSO): each held-out study defines 1-fold, yielding *n* = 20 paired observations for error metrics. For each fold and metric *m*(e.g., MAE, MAPE), we compute the paired improvement Δ_*m*_ = *m*_baseline_-*m*_Track B_. Statistical evidence for improvement is assessed using a one-sided Wilcoxon signed-rank test on Δ_*m*_(Wilcoxon, 1945), which is appropriate for paired comparisons under non-normal and heteroscedastic fold-wise errors. We do not rely on the Diebold–Mariano test because it targets serially indexed forecast-error differentials and its variance estimation and small-sample behavior are not well matched to independent, study-level fold comparisons (Diebold and Mariano, 1995; Harvey et al., 1997).

To quantify practical impact, we report (i) the Hodges–Lehmann estimate of the median improvement with a bootstrap 95% confidence interval (percentile bootstrap over folds; *B* = 10,000), and (ii) a standardized matched-pairs rank-biserial correlation effect size derived from signed ranks (Hodges and Lehmann, 1963; Cureton, 1956).

To assess the sensitivity of Track B to different descriptor blocks under sparse and correlated metadata, a block-ablation analysis was performed under the same leave-one-study-out (LOSO) protocol used for the main results. Descriptor blocks were removed one at a time (geometry-related descriptors, excitation descriptors, and materials/treatment descriptors), the model was retrained on each training fold, and fold-level performance deltas were computed relative to the full-feature model. Because fold count is limited and effects may be heteroscedastic, ablation deltas are summarized with robust location estimates and bootstrap confidence intervals across folds. In addition, two controls were used: (i) a study-stratified label-permutation negative control to verify that performance collapses toward chance under broken input–output association; and (ii) a lightweight synthetic-data experiment in which targets are generated from planted physical dependencies with controlled noise to confirm that the pipeline recovers expected relationships when they are truly present.
